# Chemosensory Dysfunction in COVID-19: Prevalences, Recovery Rates, and Clinical Associations on a Large Brazilian Sample

**DOI:** 10.1177/0194599820954825

**Published:** 2020-09-01

**Authors:** Deusdedit Brandão Neto, Marco Aurélio Fornazieri, Caroline Dib, Renata Cantisani Di Francesco, Richard L. Doty, Richard Louis Voegels, Fabio de Rezende Pinna

**Affiliations:** 1 Department of Otorhinolaryngology, University of São Paulo, São Paulo, Brazil; 2 Department of Surgery, Londrina State University, Londrina, Brazil; 3 Department of Medicine, Pontifical Catholic University of Paraná, Londrina, Brazil; 4 Smell and Taste Center, Department of Otolaryngology, Perelman School of Medicine, University of Pennsylvania, Philadelphia, Pennsylvania, USA

**Keywords:** COVID-19, chemosensory disorder, smell loss, taste loss

## Abstract

**Objective:**

Our study aimed to measure the percentage of reported olfactory or taste losses and their severity, recovery time, and association with other features in a large cohort of patients with COVID-19.

**Study Design:**

Prospective survey.

**Setting:**

Quaternary medical center and online survey.

**Methods:**

The perceived chemosensory capacities of 655 patients with confirmed COVID-19 were assessed with 11-point category rating scales (0, no function; 10, normal function). Patients were contacted in hospital, by phone calls, or by internet regarding their ability to smell or taste, and 143 were interviewed by phone 1 to 4 months later to assess the recovery of their chemosensory abilities.

**Results:**

The prevalence of self-reported olfactory, general taste, and taste quality–specific disturbances (sweet, sour, bitter, and salty) in the patients with COVID-19 were 82.4% (95% CI, 79.5%-85.3%), 76.2% (95% CI, 72.9%-79.4%), and 52.2% (95% CI, 48.3%-56.1%), respectively. The majority reported anosmia (42.9%). The presence of chemosensory symptoms was not associated with COVID-19 severity. At a median time >2 months after the onset of symptoms, rates of total and partial olfaction recovery were 53.8% and 44.7%, while complete or partial return to previous taste function was 68.3% and 27.6%. Less than 5% of the patients reported no chemosensory function improvement at all.

**Conclusion:**

The prevalence of self-reported chemosensory dysfunction is high among patients with COVID-19. Almost all patients seem to recover a significant part of their smell and taste abilities in the first 4 months after the onset of symptoms.

COVID-19, caused by the new coronavirus SARS-CoV-2, is presently responsible for hundreds of thousands deaths worldwide.^1^ Although some patients are asymptomatic, the infection may evolve, beginning with mild upper airway symptoms and progressing to severe dyspnea, the need for mechanical ventilation, and, finally, death.^2-5^

Among the reported symptoms of COVID-19 is loss of smell and taste function, the latter commonly being manifest by the loss of the flavor of foods and beverages. Initial data on the prevalence of olfactory and gustatory complaints vary from 5% to 85%.^6-9^ This chemosensory disturbance might be a valuable predictor of COVID-19 infection.^10^

Olfactory loss during upper airway infections caused by non–COVID-19 viruses is common and, on rare occasion, persists after resolution of obstruction and rhinorrhea, a condition called postinfectious olfactory dysfunction.^11-14^ Unfortunately, data on the prevalence of olfactory dysfunction in the acute phase of an upper airway infection do not exist but could clarify whether this frequency is higher in patients affected by COVID-19.

To better understand how SARS-CoV-2 affects the perception of smell and taste, we interviewed a large number of patients with confirmed COVID-19, via telephone, internet, or in person (ie, in the hospital). Our survey aimed to evaluate the percentage of reported olfactory or taste loss, severity, duration, and recovery time.

## Methods

### Study Population

Inclusion criteria for the patients of this study were (1) an age of 18 to 65 years and (2) a positive result for COVID-19 on a reverse transcriptase polymerase chain reaction test of nasopharyngeal or oropharyngeal swab specimens. A total of 545 patients were recruited by electronic contact in advertisements on social networks; 110 were inpatients at Hospital das Clínicas da Faculdade de Medicina da Universidade de São Paulo. Older patients were excluded from participation due to evidence that smell loss from other causes is common in older populations.^15^ Additional exclusion criteria were reports of preexisting olfactory or gustatory dysfunction, a history of traumatic brain injury, Alzheimer’s disease, Parkinson’s disease, epilepsy, stroke, any limb paralysis, or the use of psychiatric medications (except for depression).

### Study Design

This was a prospective observational study. The recruitment of volunteers occurred from April 10 to April 30, 2020, when the last patient was interviewed. The local ethics committee approved this study (Comissão Nacional de Ética em Pesquisa, No. 4.037.291). Eligible patients answered a questionnaire on nasal and general complaints (https://pt.surveymonkey.com/r/HVR7JW8) as well as a quality-of-life questionnaire that focused on nasal obstruction (NOSE-p3 [Nasal Obstruction Symptom Evaluation–Portuguese Version No. 3]).^16^ Individuals were recruited in 2 ways: (1) as patients hospitalized outside the intensive care unit (ICU) who tested positive for COVID-19 and agreed to participate after reading and signing the consent and (2) as those who had contact with our social media notices and volunteered to access questionnaires through the platform. For these patients, the first question requested their consent. If they agreed to participate, they continued to fill out the rest of the questionnaire. Online patients sent us a copy of the positive test result for COVID-19, with a copy of their national identity card. Forty-seven questionnaires with nonreliable responses—such as incomplete data, not sending the positive test result, or reporting contradictory answers—were excluded from the analysis.

### Measurements

A patient was considered to have an olfactory or general taste disturbance when he or she self-reported being unable to smell or taste after or at the beginning of other COVID-19 symptoms. This categorical variable was used to estimate complaint prevalence. A patient was classified as having a quality-specific gustatory deficit if he or she denied being able to differentiate among sweet, salty, sour, and bitter after the COVID-19 onset. The severity of the olfactory and taste deficits was assessed with an 11-point scale from 0 (normal function) to 10 (no function); patients scoring 10 were considered anosmics or ageusics, respectively. The magnitude of the disturbance with these symptoms was verified with a similar scale (0, no disturbance; 10, much disturbance). The scales scores 1 to 3, 4 to 7, and 8 to 10 were considered mild, moderate, and severe, respectively.^17^ The day of onset of the chemosensory complaints was calculated since the onset of the first symptom. The presence of nasal symptoms during the disease was denoted yes/no: coryza, nasal blockage, anterior and posterior rhinorrhea, epistaxis, headache, and itching of the nose.

To assess recovery rates, one of the authors (M.A.F.) interviewed 143 patients with a history of COVID-19 olfactory or taste losses between 36 and 119 days of the onset of the symptoms (median, 76; interquartile range, 66-88). He asked patients if their smell and taste capacities returned to the same level as before the SARS-CoV-2 infection; if not, the patients again rated the severity of the dysfunction.

### Statistical Analysis

The prevalence of symptoms and their severities were calculated as percentages with 95% CIs. Age and other continuous variables, such as the degree of discomfort with olfactory loss, were depicted as means and standard deviations. The association of olfactory and gustatory symptoms with demographic characteristics, as well as other nasal and general symptoms, was analyzed with logistic regression analyses with the following independent variables: patient characteristics, interval between interview and onset of symptoms in days, age, sex, race, schooling, smoking history, nasal obstruction measured by the NOSE-p3, the presence of comorbidities, development of dyspnea with COVID-19, necessity of invasive ventilation, and ICU admission. The relationship between the severity of the olfactory and gustatory complaints was verified with multiple linear regression analyses and the predictor variables for the logistic regression. In the multiple linear regression, respecting the assumption of independent data, measurements from patients interviewed again by phone calls were included in the model just once (ie, from the last interview). *P* values for all regression analyzes were adjusted for multiple comparisons by Bonferroni corrections. The level of significance was set at 0.05.

## Results

### Patients

**Figure 1.**
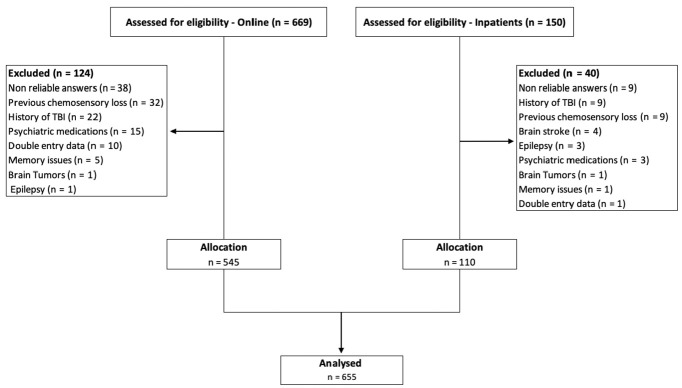
Flow diagram of patient recruitment. TBI, traumatic brain injury.

Data from 545 patients who fulfilled the online questionnaire and from 110 inpatients were included in the analyses (**Figure 1**). Patients had a mean age of 37.7 years (SD, 10.4 years), and 424 were women (64.7%). The median time between the onset of symptoms and the interview was 15 days (interquartile range, 10-24 days). Other demographic characteristics of the patients are presented in **Table 1**.

**Table 1.** Demographic and Clinical Characteristics of the Patients During the Acute Phase of COVID-19.^a^

	Total (N = 655)	Online patients (n = 545)	Inpatients (n = 110)
Age, mean ± SD, y	37.7 ± 10.4	36.4 ± 9.1	43.7 ± 13.7
Male sex	231 (35.3)	184 (33.8)	47 (42.7)
White race	493 (75.3)	438 (80.4)	55 (50)
Educational level			
Less than middle school	10 (1.5)	0	10 (9.1)
Middle school	10 (1.5)	2 (0.4)	8 (7.3)
Some high school	6 (0.9)	0	6 (5.5)
High school	61 (9.3)	27 (5)	34 (30.9)
Some college	41 (6.3)	28 (5.1)	13 (11.8)
Bachelor or higher	527 (80.5)	488 (89.5)	39 (35.4)
Smoker			
Former	105 (16)	81 (14.9)	24 (21.8)
Current	18 (2.8)	17 (3.1)	1 (0.9)
NOSE-p3 score, mean ± SD	4.9 ± 4.5	4.6 ± 4.3	6.4 ± 5.4
Comorbidities			
Yes	251 (38.3)	192 (35.2)	59 (53.6)
Hypertension	55 (8.4)	45 (8.3)	10 (9.1)
Diabetes	19 (2.9)	13 (2.4)	6 (5.5)
Asthma	11 (1.7)	8 (1.5)	3 (2.7)
Dyslipidemia	22 (3.4)	15 (2.8)	7 (6.4)
Cardiopathy	12 (1.8)	9 (1.6)	3 (2.7)
Gastroesophageal reflux	11 (1.7)	10 (1.8)	1 (0.9)
General symptoms			
Dyspnea	210 (32.1)	142 (26.1)	68 (61.8)
Fever	352 (53.7)	274 (50.3)	78 (70.9)
Digestive	288 (43.9)	248 (45.5)	40 (36.4)
Invasive ventilation	14 (2.1)	1 (0.2)	14 (12.7)
ICU admission	36 (5.5)	12 (2.2)	24 (21.8)
Interval onset symptoms and interview, median (IQR), d	15 (10-24)	16 (10-24)	13 (8-24)

Abbreviations: ICU, intensive care unit; IQR, interquartile range; NOSE-p3, Nasal Obstruction Symptom Evaluation–Portuguese Version No. 3.

a Values are presented as No. (%) unless noted otherwise.

### Prevalences

Among the 655 patients who were interviewed, 82.4% (95% CI, 79.5%-85.3%) reported experiencing olfactory dysfunction; 76.2% (95% CI, 72.9%-79.4%) complained of decreased general taste; and 52.2% (95% CI, 48.3%-56.1%) cited taste quality–specific deficits. Interestingly, the reported prevalence of these symptoms was higher in those patients who were interviewed online than in those who were interviewed as inpatients: decreased smell, 85.9% (95% CI, 82.9%-88.8%) vs 65.1% (95% CI, 56%-74.2%; *P* < .001); decreased general taste, 79% (95% CI, 75.6%-82.5%) vs 61.8% (95% CI, 52.6%-71%; *P* < .001); and taste quality–specific dysfunction, 55.5% (95% CI, 51.2%-59.7%) vs 36.1% (95% CI, 26.9%-45.3%; *P* < .001). The prevalence of the olfactory and gustatory complaints remained high during the 2-week period after the first day of the symptoms (**Tables 2** and **3**). In the logistic regression analysis, the presence of chemosensory symptoms was not associated with COVID-19 severity (ie, the necessity of invasive ventilation and ICU admission). It also was not related to age, race, and the other factors depicted in **Table 1**. The lack of associations remained when separate regression analyses were performed for inpatients and patients interviewed online.

**Table 2.** Prevalence of Nasal and Chemosensory Complaints Among Patients With COVID-19 Interviewed Online Since the First Day of Symptoms.^a^

Symptom	Total	Days 1-3	Days 4-7	Days 8-14	Days 15-55
No.	545	12	61	174	298
Olfactory dysfunction	468 (85.9)	11 (91.7)	56 (91.8)	153 (87.9)	240 (80.5)
Taste deficit	429 (78.7)	9 (75)	51 (83.6)	142 (81.6)	227 (76.2)
Nasal obstruction	117 (21.5)	2 (16.7)	22 (36.1)	57 (32.8)	36 (12.1)
Coryza	93 (17.1)	4 (33.3)	19 (31.2)	34 (19.5)	36 (12.1)
Nasal yellowish secretion	14 (2.5)	0	1 (1.6)	9 (5.2)	4 (1.3)
Epistaxis	5 (0.9)	0	0	1 (0.5)	4 (1.3)
Headache	119 (21.8)	4 (33.3)	27 (44.3)	47 (27)	41 (13.8)
Nasal itching	64 (11.7)	3 (25)	10 (16.4)	21 (12.1)	30 (10.1)
Posterior rhinorrhea	118 (21.7)	3 (25)	17 (27.9)	56 (32.2)	42 (14.1)

a Values are presented as No. (%).

**Table 3.** Prevalence of Nasal and Chemosensory Symptoms Present in Inpatients With COVID-19 Since the First Day of Symptoms.^a^

Symptom	Total	Days 1-3	Days 4-7	Days 8-14	Days 15-55
No.	110	1	20	41	48
Olfactory dysfunction	71 (65.1)	1 (100)	11 (55)	26 (63.4)	31 (64.6)
Taste deficit	73 (66.4)	1 (100)	12 (60)	28 (68.3)	32 (66.7)
Nasal obstruction	25 (22.7)	1 (100)	8 (40)	12 (29.3)	4 (8.4)
Coryza	26 (23.6)	1 (100)	7 (35)	8 (19.5)	10 (20.8)
Nasal yellowish secretion	4 (3.6)	0	2 (10)	0	2 (4.2)
Epistaxis	3 (2.7)	0	0	1 (2.4)	2 (4.2)
Headache	24 (21.8)	1 (100)	7 (35)	8 (19.5)	8 (16.7)
Nasal itching	12 (10.9)	1 (100)	3 (15)	4 (9.8)	5 (10.4)
Posterior rhinorrhea	20 (18.2)	1 (100)	7 (35)	9 (21.9)	4 (8.3)

a Values are presented as No. (%).

### Severity of Symptoms

**Figure 2.**
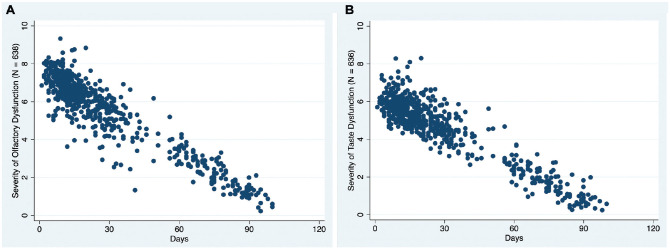
Fitted multiple linear regression shows the inverse relationship between chemosensory complaint severity as measured by visual analog scale (0, function equal to that before COVID-19 infection; 10, no function) and days after the onset of symptoms.

According to the rating scales, 39 patients experienced mild microsmia (5.9%; 95% CI, 4.1%-7.8%); 91, moderate microsmia (13.9%; 95% CI, 11.2%-16.5%); and 399, severe microsmia or anosmia (60.9%; 95% CI, 57.2%-64.7%). Fifty-one reported mild general taste loss (7.8%; 95% CI, 5.7%-9.8%); 153, moderate general taste loss (23.4%; 95% CI, 20.1%-26.6%); and 298, severe or total general taste loss (45.5%; 95% CI, 41.7%-49.3%). In addition, 281 (42.9%; 95% CI, 39.1%-46.7%) considered themselves anosmic and 137 (20.9%; 95% CI, 17.8%-24%) ageusic. Severity of the smell and taste losses diminished over days (smell: β = −0.07, per day [95% CI, −0.08 to −0.06], *P* < .001; taste: β = −0.06, per day [95% CI, −0.07 to −0.05]; **Figure 2**) and was not related to age, sex, race, educational level, smoking habit, nasal obstruction, dyspnea during COVID-19 symptomatology, and the necessity of invasive ventilation or ICU admission.

### Recovery of Olfactory and Gustatory Dysfunctions

After a median 76 days from the beginning of COVID-19 symptoms, 77 of the 143 patients with an olfactory deficit (53.8%; 95% CI, 45.6%-62.1%) reported total recovery; 44.7% (95% CI, 36.5%-53%), partial recovery; and 1.4% (95% CI, 0%-3.3%), no recovery. In relation to taste dysfunction, 68.3% (95% CI, 59.9%-76.6%) of the patients with this complaint reported total recovery; 27.6% (95% CI, 19.6%-35.6%), partial recovery; and 4.1% (95% CI, 0.5%-7.6%), no improvement. As seen in **Figure 2**, despite the fact that almost half of the patients continued to have some chemosensory dysfunction, it was much less intense than at the beginning of the disease. Interestingly, in the period studied, women had 80% less chance to totally recover olfactory function as compared with men (odds ratio, 0.2; 95% CI, 0.06-0.51; *P* = .001). This association was not found for taste.

## Discussion

This study found a high prevalence of self-reported severe olfactory and general taste disorders in a large sample of hospitalized and nonhospitalized patients with COVID-19. Importantly, most patients reported difficulties in experiencing or differentiating among the basic taste qualities of salty, bitter, sweet, and sour, strongly suggesting that the chemosensory capacities of the taste bud–mediated taste system are affected in this disease. The reported deficits occurred early in the course of the disease and, in a few cases, were reported as the first or unique disease symptom. Chemosensory complaints were not associated with the demographic or clinical characteristics of the patients, such as the necessity of invasive ventilation or ICU admission. There was a significant decrease in the severity of these symptoms with time. More than half of the patients reported total recovery of their smell and taste function. Interestingly, a lower percentage of women reported total recovery than men.

It is noteworthy that the prevalence of chemosensory dysfunction was higher in individuals who completed the questionnaires online than in those who did so in the hospital. There are several reasons why this may have occurred. First, the inpatients may not have noticed their chemosensory deficit in light of their other disease symptoms, such as dyspnea. Second, there may have been a sampling bias for the online survey, specifically oversampling persons whose function had not improved over time. Third, the online patients may have been more affected by media attention, which is positively correlated with the number of self-reported symptoms.^10^

It is of interest that the onset of the smell dysfunction was, on average, 3.5 days after the start of other symptoms. Seventy patients (12.8%) reported smell impairment as unique or among their first symptoms or as occurring with them. These data are close to the 11.8% found by Lechien et al and reinforce the importance of this clinical finding for the early diagnosis of the disease.^18^

The majority of our patients presented with severe smell and taste losses, with a high proportion considering themselves anosmics and ageusics. This is in accordance with the first study of the Global Consortium for Chemosensory Research (https://gcchemosensr.org), which revealed significant COVID-19–related reductions in self-reports of smell, taste, and chemesthesis (chemical sensitivity of the skin and mucous membranes).^19^ It is also in accord with the findings of a recent study in which olfactory function was empirically measured in patients with COVID-19.^20^

Yan et al found that patients with COVID-19 and anosmia are 10 times less likely to be hospitalized than their normosmic counterparts. Such observations suggest that severe chemosensory dysfunction is unrelated to measures typically related to hospitalization and that anosmia or severe hyposmia reflects milder forms of the disease.^21^ However, in our study, 36 patients (5.5% of the total sample) reported having been admitted to the ICU, of which 24 reported taste and smell losses. This indicates a 66.6% prevalence rate of chemosensory complaints in this more severe group of patients with COVID-19. We found no association between the presence of any chemosensory loss and the need for more invasive ventilation or admission to the ICU. These findings corroborate an Iranian study in which there was no apparent association between scores on the University of Pennsylvania Smell Identification Test and the severity of the disease.^20^

Regarding the recovery of smell and taste function, some studies suggest that a significant portion of patients with COVID-19 show an improvement in such function coincident with or immediately after clinical resolution of the disease.^21-23^ In the present study, the rates of complete self-reported smell and taste recovery at a median 76 days after the onset of symptoms were 53.8% and 68.3%, respectively. Furthermore, >95% of the patients achieved a significant improvement of these functions. These data suggest that the sensorineural damage seems to be largely reversible and that a longer follow-up period is needed to confirm whether complete recovery occurred in the majority of our sample. The monitoring of these patients should be carried out to identify the rate of patients with persistent complaints. Future studies with validated smell and taste tests should clarify the rate of recovery of these senses.

Numerous studies have found that women outperform men on a variety of olfactory tests.^24,25^ Thus, in the short term, a persistent minimal deficit in the chemosensory function attributed to SARS-CoV-2 infection could probably be more perceptible to them, justifying their lower percentage of total recovery when compared with men.

This study has strengths and weaknesses. Among its strengths are the use of rating scales to establish relative degrees of symptom severities, the sampling of a large number of well-documented patients with COVID-19 via a detailed questionnaire, and the evaluation of hospitalized and nonhospitalized patients. The major limitations are twofold. First is the possibility of sampling or participation bias. Our study was based on a survey announced on social media, and the cover image identified the study as being about COVID-19 and smell changes. This may have motivated more patients with smell complaints to answer the questionnaire, resulting in an overestimation of prevalence. The other limitation is shared by the majority of studies on this topic—namely, the use of self-reports. Self-reports likely underestimate the true prevalence of chemosensory losses, as is known for non–COVID-19 cases since many patients with such losses are unaware of their deficits until formal testing.^20^ For example, Moein et al measured smell function in 60 patients with COVID-19 using the 40-item University of Pennsylvania Smell Identification Test and found a prevalence of 98%,^20^ a rate much higher than ours and other self-report–based studies.^10,18^ The discrepancy between the loss of smell by self-report and that measured with validated quantitative tests should be addressed in future studies evaluating larger sample sizes with olfactory tests.

## Conclusion

This study has shown a high prevalence of self-reported chemosensory deficits associated with equally high severity of symptoms. Smell and taste complaints began, on average, 3 days after the onset of the other COVID-19 symptoms. Olfactory and taste reductions were the first symptoms in 70 (12.8%) and 62 (11.8%) patients, respectively. Chemosensory deficits were the unique symptom of COVID-19 in 19 patients (2.9%). Almost all patients totally or partially recovered their taste and smell as before COVID-19, but further data with psychophysical data are mandatory to confirm these self-reported findings.
